# Analyzing Multidimensional Poverty and Its Determinants in Ethiopia: A Micro-level Analysis at Debre Tabor City Administration

**DOI:** 10.12688/f1000research.159139.1

**Published:** 2025-02-13

**Authors:** Yismaw Ayelign Mengistu, Bantalem Sinishaw Mekonen, Nega Assefa Aynalem

**Affiliations:** 1 Assistant Professor, Economics, Debre Tabor University, Debre Tabor, Amhara, 272, Ethiopia; 2 Lecturer, Economics, Kabridahar University, Kebri Dahir, Somali, 250, Ethiopia; 3 Lecturer, Economics, Debre Tabor University, Debre Tabor, Amhara, 272, Ethiopia

**Keywords:** Alkire and Foster; MPI; MPI decomposition; multidimensional poverty; ordered logistic regression

## Abstract

**Background:**

Poverty remained pervasive in affecting households particularly in developing countries like Ethiopia.

**Purpose:**

The main target of this study was to estimate the multidimensional poverty index of households in Debre Tabor city along with the analysis of determinants of multidimensional poverty.

**Method:**

The researchers selected 394 households from six Kebeles using a stratified random sampling technique. The Alkire and Foster dual cut-off method of measuring poverty was employed to estimate the multidimensional poverty index and ordered logistic regression was used to analyze the effects of determinants of multidimensional poverty. Five dimensions of poverty namely, income, education, health, living standards, and energy use were incorporated in measuring it.

**Results and conclusion:**

The result revealed that 59% of households were multidimensional poor with a poverty cut-off of 33%. The multidimensional poor households were underprivileged in 54% of weighted indicators. The energy use dimension was a serious contributor to the incidence and severity of multidimensional poverty. In addition, poverty incidence by illiterate-headed household heads, with single marital status, and casual wage worker-headed households was higher relative to their counterparts.

**Policy recommendation:**

We strongly recommended that education must be extensively provided not only to children but also to adults; females must be promoted to participate in business activities, credit must be expanded with an excellent follow-up, and households should participate in existing cooperatives.

## 1. Introduction

Every human being in the world faces the multifaceted threat and challenge of poverty at the levels of the economy, society, politics, and environment ( Kvaratskhelia, 2019). Poverty is defined as a lack of resources necessary to maintain a standard of living at an economic level. It also refers to a state in which an individual lives under unsafe circumstances, lacks agency in society, and has no voice. Beyond its financial component, poverty is defined as a household’s inability to obtain standard housing, child mortality, inadequate access to clean water and sanitation, years of education relevant to children’s attendance at school, malnourishment, lack of fixed assets, and lack of clean energy for cooking ( WB, 2018; Alkire et al., 2021).

The inability to buy a home of their own limits the options of the poor; instead, they are more likely to rent poorly maintained homes with inadequate drainage, ventilation, and sanitary facilities ( Haider & Kumar, 2018). Additionally, poverty has several detrimental effects on household members’ social relationships. They are embarrassed and furious and are unable to maintain their regular social circles and friendships ( Mood & Jonsson, 2016).

According to Ethiopian Demographic and Health Survey (EDHS) (2021), the UNDP and OPHI (2021), 68.7% of Ethiopians are multidimensional poor. On average, devoid of ten indicators of multidimensional poverty, including years of schooling, child attendance, nutrition, child mortality, access to electricity, clean drinking water, improved sanitation, quality of housing, and assets.

With the exception of the Tigray Region, Addis Ababa, and Dire Dawa, the regional breakdown of Ethiopia’s multidimensional poverty level reveals that the percentage of the population living in multidimensional poverty is higher than that of the country’s overall multidimensional poverty rate. Somalia regional state was the area with the highest multidimensional poverty rate (90%). The second most multidimensional impoverished region was Afar at 84.7%). Ethiopia’s third- and fourth most multidimensional impoverished areas are Oromia (71.5%) and Amhara (69.3%), respectively ( OPHI & UNDP, 2021).

A number of one-dimensional or monetary methods, including the Foster-Greer-Thorbecke method ( Debeli and Endegena, 2019; Dagim et al., 2020), the basic needs approach ( Melese et al., 2017; Debeli, 2019), and food energy intake ( Tamirat and Sivakumar, 2020), have been used in empirical studies to measure poverty in Ethiopia. This suggests that most studies on poverty measurement in urban areas rely on only one dimension.

However, Mekonnen and Heshmati (2021) pointed out that there is a risk of underestimating poverty when utilizing a one-dimensional measure approach to quantify poverty. Thus, the multidimensional method of calculating poverty level developed by Alkire and Foster (2011a, 2011b) was used in this study.

Furthermore, to quantify the impact of determinant variables on poverty level, earlier studies employed binary response regression models (logit and probit). Therefore, households were divided into two categories based on their level of poverty: poor and non-poor, according to earlier research. There must be more ways to alleviate poverty than just this one because not all households with a poverty status below the poverty line and not all households with a poverty status above the poverty line are equally impoverished. The primary issue with this approach is that it may lead to ineffective focus on poverty alleviation by dividing the population into two groups.

This indicates that while the extremely impoverished population needs more attention, the population whose economic position is below the poverty line cannot be equally addressed by agencies working to alleviate poverty. Similarly, those whose economic standing is above the poverty line cannot be disregarded in the same way as those who are just above the line are more susceptible to poverty and therefore require greater care. Therefore, further household classification and a suitable econometric model is needed to determine how socioeconomic variables affect multidimensional poverty levels.

Four non-overlapping multidimensional poverty level groups were created for homes in this study. According to the multidimensional index, these fall into four categories: extremely multidimensional poor, somewhat multidimensional poor, susceptible to multidimensional poverty, and not impoverished. The deprivation score determined for every household served as the basis for such a classification. Therefore, to evaluate the effects of various socioeconomic variables on these four groups of multidimensional poverty levels in the Debre Tabor city administration, an ordinal logistic regression model was adopted in this work.

The main objective of this study was to estimate the multidimensional poverty index (MPI) of the households in Debre Tabor City administration as decomposed by indicator and determinants factors of multidimensional poverty.

The remainder of this paper is organized as follows. Section two is devoted to a review of the literature. Section three deals with the methods and materials used in this study. The last two sections present the results, conclusions, and recommendations of this study.

## 2. Literature

Poverty refers to a lack of income and sufficient resources to sustain living conditions. It manifests as hunger and malnutrition, ill-health, limited access to education, high mortality rate from illness, sub-standard housing, insecure environments, and social marginalization. It is also characterized by limited involvement in the decision-making of the social, cultural, and political affairs of a society ( UN, 1995; WB, 2000).

### 2.1 Measuring poverty: Multidimensional approach to measuring poverty

The Alkier–Foster approach is a well-known and used method of multidimensional poverty measurement. However, the Alkire-Foster method is widely used because other methods have basic drawbacks, such as they are not compatible with making comparisons across regions or countries ( Alkire et al., 2015).

Alkire and Foster (2007 revised in 2008) have developed a counting approach to measuring multidimensional poverty which dual cut-off approach to measuring multidimensional poverty and it is being utilized since 2010 to estimate the MPI of different countries by UNDP and OPHI. The dual cut-off has two steps; the first is the identification step and the second is the aggregation step ( Alkire & Foster, 2011a).

On the behalf of UNDP and OPHI Alkire et al. (2010) have prepared the dimensions and their respective indicators of poverty that are being used to estimate the multidimensional poverty index of different countries globally.

**Dimensions and indicators of global multidimensional poverty index**

The first dimension of poverty is education, which has two indicators: years of schooling and school attendance. According to Alkire et al. (2010), deprivation in years of education occurs when at least one household member has completed 6 years of schooling. Alternatively, this occurs if not all household members attend school. The deprivation of school attendance exists if school-aged children do not enter school.

The second dimension relates to health and contains nutrition and child mortality as poverty indicators. When there is a death of any less than or equal to five years of age in the household and the nutritional information of a family is malnourished, deprivation in the health dimension of poverty exists. Nutritional information was measured using body mass index.

The third dimension is living standards, in which there are six poverty indicators. The indicators are cooking fuel, drinking water, sanitation, electricity, housing, and asset ownership. If the household uses animal dung or firewood instead of electricity for cooking purposes, it is considered poor in cooking fuel. Households are said to be impoverished in drinking water when its source is from unimproved locations such as ponds, unprotected dug wells, unprotected rivers, and spring water. The household is also poor in electricity if it does not have access to electric energy in its house at all. In addition, the household is impoverished in housing quality if its house is made of a traditional floor, wall, and roof material such as sand, soil, animal dung floor, grass roof, and bamboo or wood wall, respectively. Poor sanitation exists when there is no modern or improved sanitation system. That is, it occurs when the household uses open defecation, unventilated latrine toilets, and so on. In addition, the household is considered a poor asset if it does not own a radio, television, mobile phone, earth-line phone, refrigerator, motorbike, motorcycle, car, or computer.

**Alkire-Foster counting approach to estimating multidimensional poverty index**

Measuring poverty using Alkire-Foster’s (2011a) method follows a dual-cut-off approach. In this method, there are stages, which are the identification and aggregation stages. To accomplish the first stage of MPI, different types of cut-off are required. The First is the deprivation cut-off, which is the process of setting the minimal value of an indicator of poverty below which a household or any unit of analysis is said to be deprived in that dimension or indicator. After the deprivation cutoffs are set, the deprivation scores of each individual or another unit of analysis will be prepared. The second cutoff was poverty cutoff. It is a line that demarcates poor and non-poor individuals. It is mostly determined by policymakers or researchers based on the realities surrounding it. For example, UNDP and OPHI use 0.33 as a poverty cut-off in global MPI.

According to the authors, the aggregation stage follows the identification stage, and provides information on the multidimensional levels of a country or region. The multidimensional headcount ratio (H), depth or intensity of multidimensional poverty (A), and adjusted headcount ratio (MPI) are the most widely used. The first case informs the proportion (percentage) of the population that is multidimensional poor. The second shows how the multidimensional poor are poor. In other words, it shows the percentage of weighed indicators of poverty in which the multidimensional poor were deprived. MPI measures the percentage of indicators potentially depriving the total potential deprivations that could be experienced by society.

### 2.2 Empirical literature: Determinants of multidimensional poverty

In South Africa, the higher income of the household head and male head of household is negatively related to poverty while the household size and marriage are negatively related to poverty ( Mdluli and Dunga, 2021).

According to Lekobane and Seleka (2016), poverty is determined negatively by the higher level of education and permanent employment and self-employment status of the head of household in Botswana. But large household size is found to increase the probability of being poor.

A study by Teshome and Quaicoe (2014) in Ethiopia revealed that variables such as dependency ratio, family size, household head being female, marital status (being married), and living in rural areas are positively correlated with the probability of being poor. However, the number of working members in the household, agricultural land holdings, family size squared, age of household head, having completed elementary education, having completed secondary education, having college education and above, being self-employed, and being household head employed in the formal sector are negatively correlated with the probability of being poor.

Another study was conducted in Southern Nations, Nationalities, and Peoples’ Region, Ethiopia education, saving habits and singleness of household heads, and availability of electricity for lighting and cooking are found to be significant variables to decrease the probability of being poor ( Mohammed, 2017).

In Ethiopia, many studies hav been conducted on the determinants of poverty. Among them, a study by Feleke et al. (2020), the education level of household heads and time spent on income-generating activities affected negatively poverty in the Kuyu district whereas distance from the market contributes positively to the problem of poverty.

Another study was conducted in Southern Nations, Nationalities, and Peoples’ Region, Ethiopia education, saving habits and singleness of household heads, and availability of electricity for lighting and cooking are found to be significant variables to decrease the probability of being poor ( Mohammed, 2017).

Study in Debre Markos City by Debeli (2019) using binary logit model explained that age, sex, marital status, education, occupation, income, number of working hours, and household health status were negatively related to the probability of being poor.

A household with an old-age leader and a female leader has more probability fall into multidimensional poverty in Boorana ( Galgalo et al., 2021).

Family size, household education, contact with extension services, and access out of household labor work, are variables negatively associated with probability of being multidimensional poor households of Dega Tembien in Tigray regional state while dependency ratio and credit access worsen it ( Desawi et al., 2021).

Gedefaw (2021) found that family size, access to credit service, and distance from market centers, had increased the probability of households in North Wollo Zone being multidimesnionally poor.

According to a study by Dagim et al. (2020), higher education by head of the household, owning a house had negative relationship with the probability of households being poor while large family size and divorce had positive relationship with the probability of households being poor

In summary, the probability that households are poor is highly positively correlated with large family size, lower education level, higher dependency in the household, unemployment, and divorce, while it is negatively correlated with credit access, household income, higher education level, saving, access to extension services, and house ownership.

## 3. Methods

### 3.1 Sampling design and sample size

Proposal for the study was evaluated in July, 2023 and actual data collection started in October, 2023.

In this study, a stratified random sampling technique has been employed to provide the proportional chance of being selected by households of each Kebeles of Debre Tabor city administration to the size of each household. The total number of households in the city was 28,036 ( DTTAO, 2021). Using Yamane’s (1967) formula, illustrated in Israel (1992), the sample size of this study was determined as follows:

$$n=\frac{N}{1+N\left(e^{2}\right)}$$ (1)

where n is the sample size, N is the total household, and the level of precision is specified as 5% in this study.

$$n=\frac{28,036}{1+28,036\left(0.05^{2}\right)}=\frac{28,036}{71.09}=394.3\approx 394$$

Thus, 394 households were used as the sample from which the data for this study were collected. Stratified sampling was applied to allocate 394 households to the Kebeles of the Debre Tabor city in proportion to the number of households in each Kebele. The proportional allocation of this sample size to each stratum $n_{i}=n*p_{i}$ where n _i_ is the sample from the i ^th^ Kebele, n is the sample size, and p _i_ is the proportion of households in Kebele i to total households ( $N_{i}/N$ ) ( Kothari, 2004).

### 3.2 Data source, type, and collection methods

This study used a questionnaire to obtain the necessary primary data. This study used a structured questionnaire. It was translated to the local language Amharic and administered by the researcher by contacting the respondent physically and explaining questions. Using this method of data collection, cross-sectional data were collected on indicators of multidimensional poverty and the determining variables of multidimensional poverty in Debre Tabor City.

### 3.3 Methods of data analysis

This study used descriptive statistics, the method of estimating the multidimensional poverty index, and econometric data analyses. The dual cut-off approach to measuring multidimensional poverty was employed to estimate the multidimensional poverty index. Finally, an ordinal logistic regression model was applied to analyze the determinants of multidimensional household poverty.

**3.3.1 Econometric model specification**

The ordered logistic regression model was chosen because the total number of households was divided into four categories based on their deprivation score ( $Y_{i}^{*}$ ). Based on the values of the deprivation score ( $Y_{i}^{*}$ ), the household categories are severely multidimensional poor households, moderately multidimensional poor households, vulnerable to multidimensional poverty, and non-multidimensional poor households. The model was then specified as follows ( Gujarati and Porter, 2009; Liu, 2016).

$$Y_{i}^{*}=\sum_{n=1}^{k}\beta_{n}X_{\text{in}}+u_{i}$$ (7)

Where $Y_{i}^{*}\;$ is unobserved, and is a latent variable. In this study, $Y_{i}^{*}$ is the weighted deprivation score of a household, which is calculated using Equation (2); β s are coefficients, Xs are the set of explanatory variables, and $u_{i}$ is the error term. There were 394 independent households (or observations), and they faced four ordered categories, which are stated as follows:

$$Y_{i}=0,\text{if}\;Y_{i}^{*}\leq \alpha_{1}\;\text{Non‐multidimensional poor}$$

$$Y_{i}=1,\text{if}\;\alpha_{1}<Y_{i}^{*}\leq \alpha_{2}\;\text{Vulnerable to}\;\mathrm{be}\;\text{multidimensional poor}$$

$$Y_{i}=2,\text{if}\;\alpha_{2}<Y_{i}^{*}\leq \alpha_{3}\;\text{Moderately multidimensional poor}$$

$$Y_{i}=3,\text{if}\;Y_{i}^{*}>\alpha_{3}\;\text{Severely multidimensional poor}$$

Where *Y*_*i*_ is a household’s multidimensional poverty level whose multidimensional poverty status would be in one of the four categories, and $\;\alpha_{1}$ , $\;\alpha_{2}$ and $\alpha_{3}$ are thresholds by which the non-overlapping demarcation was made for these categories. In this study, these thresholds are different levels of weighted deprivation scores experienced by households. That is $\alpha_{1}=0.20$ , $\;\alpha_{2}=0.33$ and $\;\alpha_{3}=0.50$ .

The estimation of the model requires determining the odds ratio. To find the odds ratio, the cumulative probability distribution of $Y_{i}$ is required because it is the ratio of the probability of being in or below category and the probability of being above category *j*. The cumulative probability distribution is given by equation 6.

$$P\left(Y_{i}\leq j\right)=P\left(\sum_{n=1}^{k}\beta_{n}X_{\text{in}}+u_{i}\leq \alpha_{j}\right)=P\left(u_{i}\leq \alpha_{j}-\sum_{n=1}^{k}\beta_{n}X_{\text{in}}\right)$$ (8)

Having this cumulative probability distribution, the probabilities being at or below a category j is given by equation 7; the probability of being above category *j* is given by equation (9) and the odds ratio which is the ratio of the two complementary probabilities is given equation (10).

$$P\left(Y_{i}\leq j|X\right)=\frac{1}{1+e^{-z_{i}}}$$ (9)

$$P(Y_{i}>j\left|X\right)=1-P(Y_{i}\leq j\left|X\right)=1-\frac{1}{1+e^{-z_{i}}}=\frac{1}{1+e^{z_{i}}}$$ (10)

$$\text{odds}\;\left(Y_{i}\leq j|X\right)=\frac{P\left(Y_{i}\leq j|X\right)}{P\left(Y_{i}>j|X\right)}=\frac{P\left(Y_{i}\leq j|X\right)}{1-P\left(Y_{i}\leq j|X\right)}=\frac{\frac{1}{1+e^{-z_{i}}}}{\frac{1}{1+e^{z_{i}}}}=e^{z_{i}}$$ (11)

Where $z_{i}=\alpha_{j}-\sum_{n=1}^{k}\beta_{n}X_{\text{in}}$

Taking natural logs on both sides of Equation (9) gives the following model, which is linear in parameter and makes it easy to interpret the coefficients.

$$Y_{i}=\ln\left[\frac{P\left(Y_{i}\leq j|X\right)}{1-P\left(Y_{i}\leq j|X\right)}\right]=\ln e^{z_{i}}=z_{i}=\alpha_{j}-\sum_{n=1}^{k}\beta_{n}X_{\text{in}}$$ (12)

Where *j* = 0, 1, 2, 3

This would provide four orders of logit or log-odds, all of which have the same explanatory variables but different intercepts (cut points). In this study, there were four categories with three thresholds.

**3.3.2 Diagnostic tests**

Cross-sectional data usually face multicollinearity and heteroscedasticity problems ( Gujarati and Porter, 2009), multicollinearity and heteroscedasticity should be checked and resolved if they appear. However, unlike ordinary least squares and binary logistic regression models, the post-estimation of ordered logistics does not support the techniques of testing multicollinearity and heteroscedasticity problems. Therefore, the model was estimated using robust standard errors to remove heteroscedasticity problems.

The other diagnostic test was the proportional odds ratio/parallel regression assumption of an ordered regression model. The assumption is stated that the coefficients of the explanatory variables are equal across ordinal categories ( Liu, 2016). The Brant test was used to check whether this assumption holds ( Brant, 1990). The intuition of the test is that the model is dichotomized many times to form different binary logits, and then the coefficient across these binary logit models will be equal. The assumption would be violated if the result of the Brant test shows a significant p-value of the chi-square test.

Finally, the overall significance of the model is the required characteristic of the econometric estimations. In this study, the log-likelihood ratio test statistic, which follows a chi-squared distribution, was used as a measure of the robustness of the model.

## 4. Results and Discussions

### 4.1 Analysis of multidimensional poverty

Depending on the weighted deprivation score in the indicators of poverty, respondents were divided into four categories: SMP, MMP, VMP, and NMP.

As is depicted in Table 4.1, only 27.6% of households were NMP, which means that those households were deprived of less than 20% of the 14 indicators of multidimensional poverty used in this study. In addition, 13.45% were also NMP, but they were VMP. Households in this poverty group were deprived of 20%-33% of indicators of multidimensional poverty. 27.41% of households were MMP, which means that they were underprivileged in 33-50% of indicators of multidimensional poverty. The remaining 31.98% of households had SMP. These households are deprived of more than 50% of indicators of multidimensional poverty. SMP households were deprived of at least seven of the 14 indicators of multidimensional poverty.

**Table 4.1.** Multidimensional poverty category.

Multidimensional poverty category	Frequency	Percent	Cum.
NMP	107	27.16	27.16
VMP	53	13.45	40.61
MMP	108	27.41	68.02
SMP	126	31.98	100.00
Total	394	100.00	

Source: Authors’ Computation.

### 4.2 The multidimensional poverty index of Debre Tabor city administration

The results in Table 4.2 shows that the multidimensional poverty incidence (headcount ratio) in Debre Tabor city administration was 59.4%, which means that 59.4% of households are multidimensional poor and could not meet at least 33% (poverty cut-off ) of the 14 indicators of poverty that were incorporated in this study. Accordingly, the households in Debre Tabor city are in acute multidimensional poverty. The intensity (depth) of multidimensional poverty was 54.9%. This means that the multidimensional poor households were deprived of 54.9% of weighted indicators of poverty, or these multidimensional poor households need to have 54.9% of indicators of poverty to pass the poverty cut-off. The adjusted headcount (MPI) in the Debre Tabor city is 0.326, which is stated as the total households (both multidimensional poor and multidimensional poor) were deprived in 32.6% of weighted indicators.

**Table 4.2.** Multidimensional poverty index (MPI).

Measures	Value	Standard errors	95% confidence interval	
Headcount (H)	59.4%	0.025	0.545	0.642
Intensity (A)	54.9%	0.01	0.529	0.568
Adjusted headcount index (MPI)	0.326	0.015	0.297	0.355

Source: Authors’ Computation.

The results revealed that the multidimensional poverty incidence, intensity, and adjusted headcount ratio are more severe in Debre Tabor city than in the respective values at the national urban level ( OPHI, 2021). The multidimensional poverty incidence, the intensity of multidimensional poverty, and adjusted headcount (MPI) were 39.2%, 47.6%, and 0.187, respectively.

### 4.3 Percentage contribution of each indicator and dimensions to MPI

**Figure 4.1.**
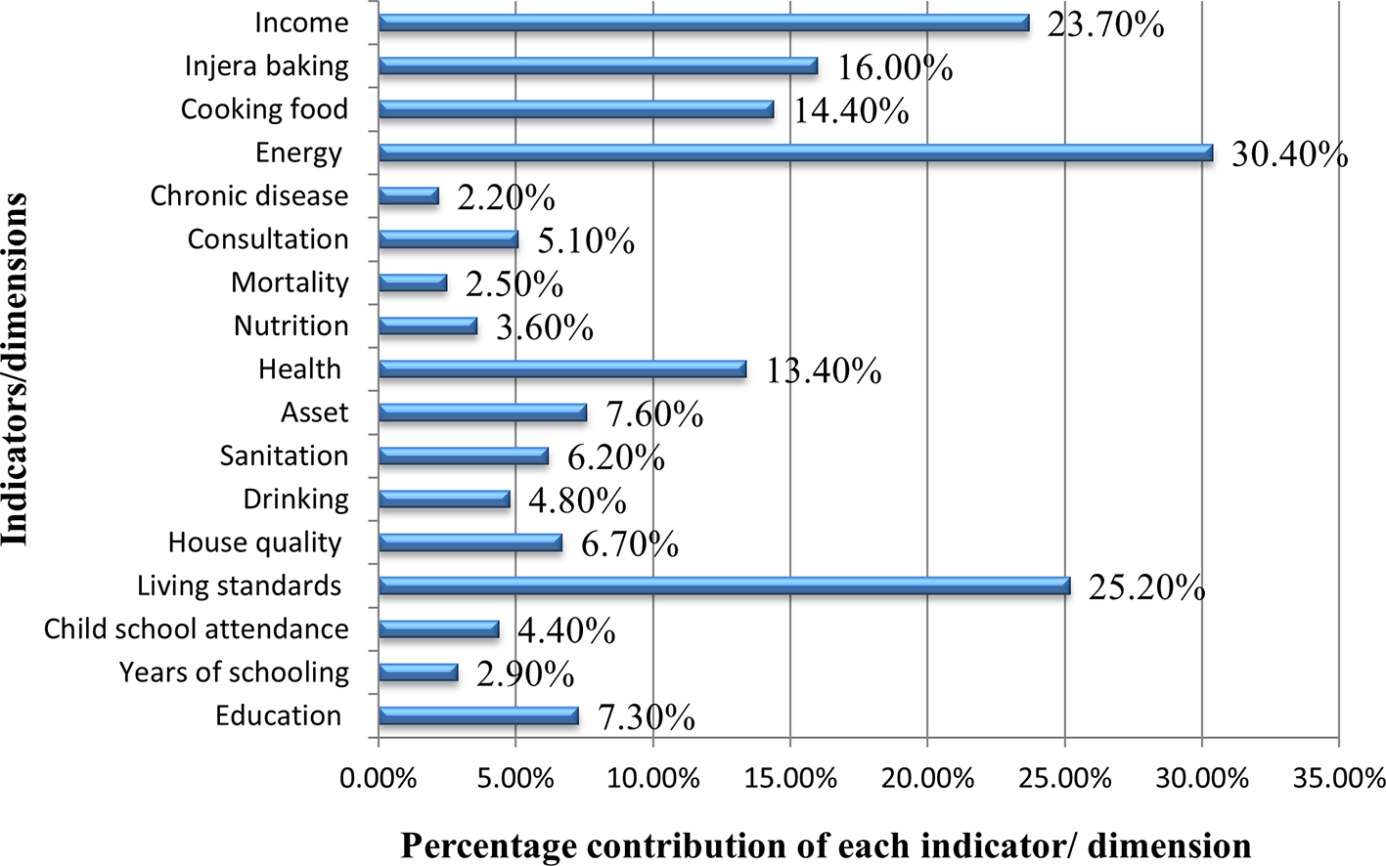
Percentage contribution of each dimension/indicator to MPI.

Figure 4.1 presents the percentage contribution of each indicator and dimension to the MPI. Education as a dimension contributed 7.3% to MPI. Years of schooling and child school attendance each contributed 2.9% and 4.4% to MPI respectively. Indicators of living standards contribute 25.5% to the MPI jointly. Separately, the quality of living houses contributed 6.7%, drinking water contributed 4.8%, sanitation contributed 6.2%, and asset ownership contributed 7.6% to the MPI. Health dimension also contributes 13.4% to the MPI in general and particularly, nutrition contributes 3.6%, child mortality contributes 2.5%, consultation contributes 5.l%, and chronic disease contributes 2.2% to the MPI.

The greatest contribution to the MPI came from energy which shares 30.4% as a dimension and cooking meals contributes 14.4 %), and baking *injera* contributes 16%). Finally, income stands up as a dimension and, at the same time, as an indicator itself, contributes 23.7% to the MPI.

Thus, the dimensions of poverty that contribute largely to the MPI of Debre Tabor city in the order of their percentage are energy sources for cooking and living standards.

### 4.4 Decomposition of MPI by sub-group population

Table 4.3 presented the decomposition of MPI by gender of household head, and the result shows that 67.9% of female-headed households are multidimensional poor, while 54.75% of male-headed households are multidimensional poor. This result indicates that female-headed households are multidimensional poorer than male-headed households. The depth of multidimensional poverty is also greater for female-headed households.

**Table 4.3.** MPI by gender of household head.

Measures	Male headed	Female-headed	Total
Headcount ratio (H)	54.7%	67.9%	59.4%
Intensity (A)	55.03%	54.49%	54.9%
Adjusted headcount ratio (MPI)	0.301	0.370	0.326
Population share (%)	64.5	35.5	

Source: Authors’ Computation

**Figure 4.2.**
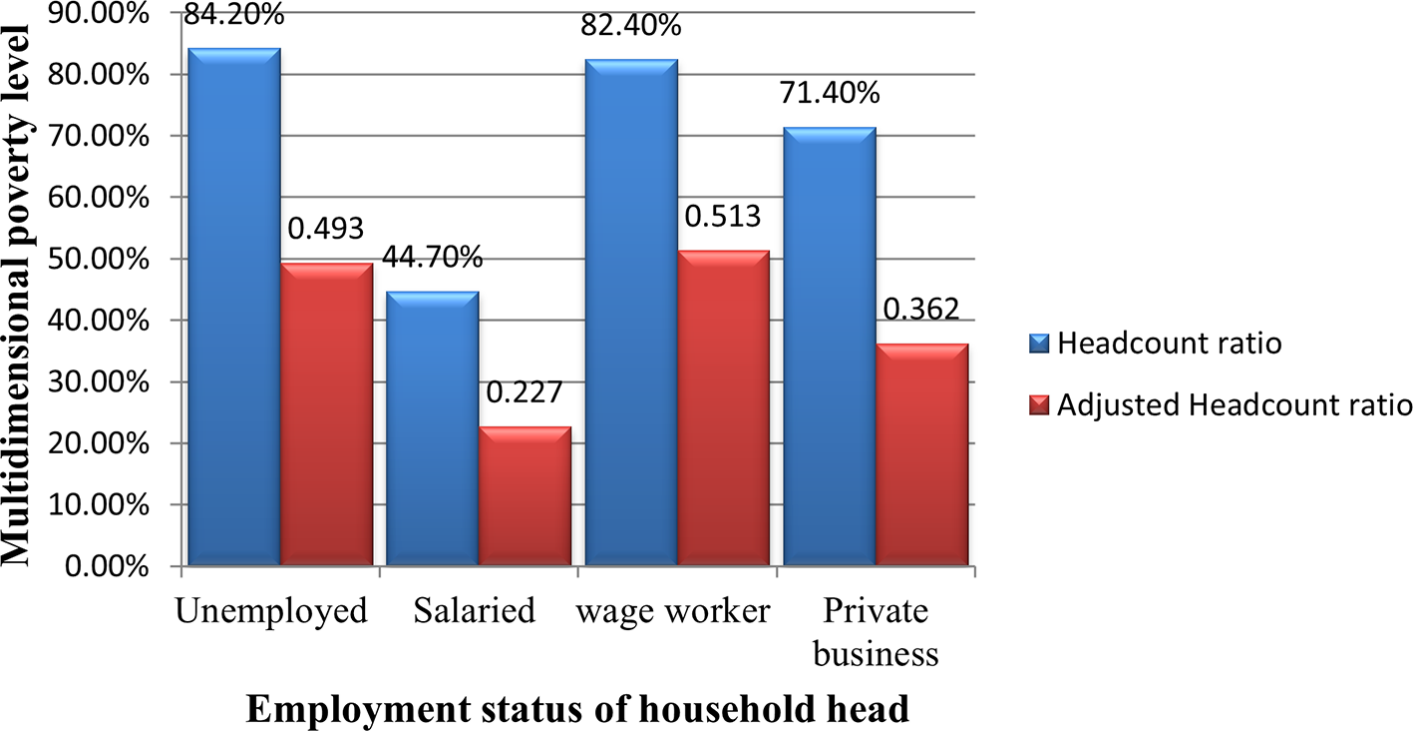
MPI by employment types of household head.

As per Figure 4.2, in the Debre Tabor city, households that have unemployed heads are the multidimensional poorest, which is 84.2% of these households are multidimensional poor. The Households that have daily wage worker heads are the second multidimensional poorest households a multidimensional poverty incidence of 82.4%. Households with heads that have a private business (self-employed) are the third most multidimensional poor. The least multidimensional poor households are those that have permanently salaried household heads a multidimensional headcount ratio of 44.7%. According to the results, households with heads of employed permanent salaries are in a better position of multidimensional richness.

**Figure 4.3.**
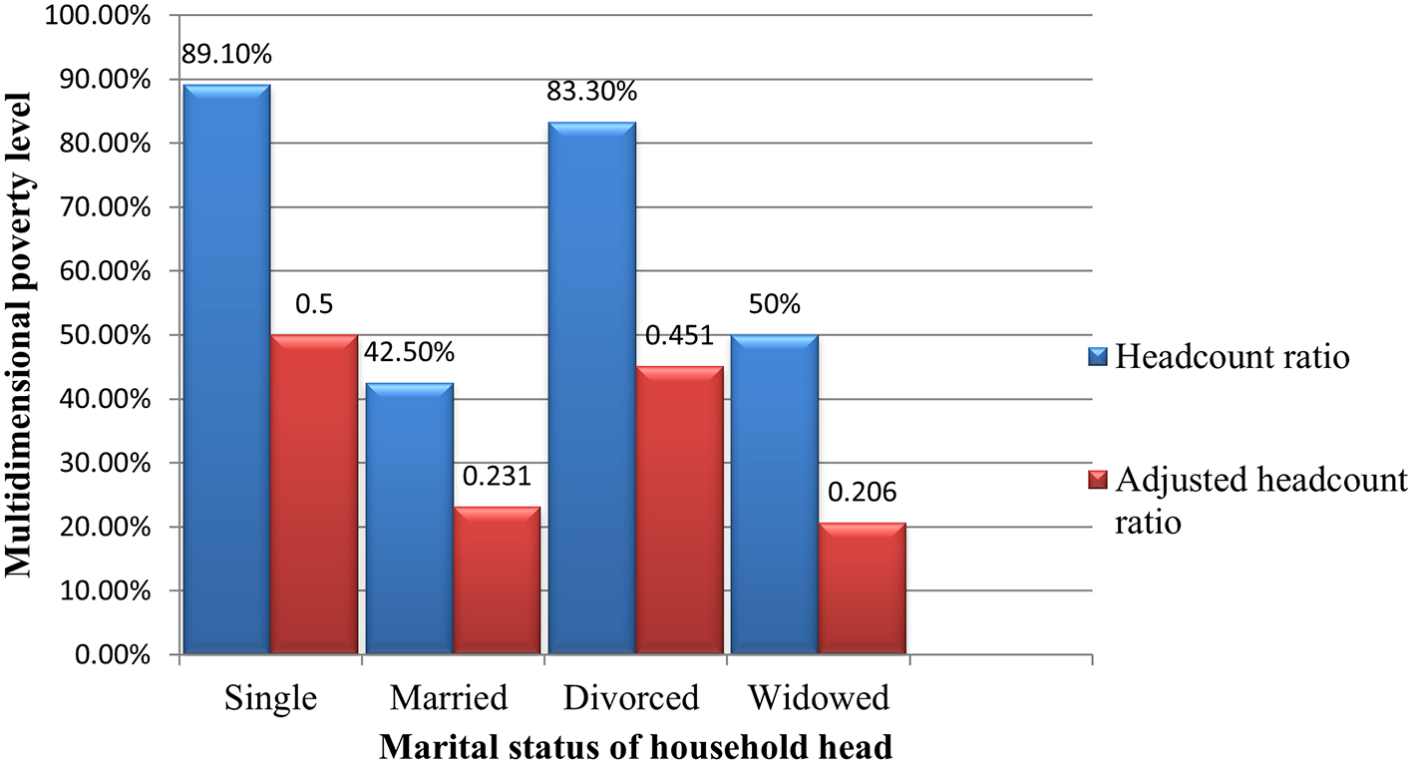
MPI by the marital status of household head.

Figure 4.3 presented the decomposition of MPI in Debre Tabor city based on the marital status of the household head. The multidimensional headcount ratio for the single-headed households was 89.5%; for the married head was 45.5%; for divorced 83.3%, and for widowed 50%. This result indicates that staying unmarried and divorced after marriage are caused by a higher multidimensional poverty level. By contrast, households whose heads are married are less multidimensional poor.

### 4.5 Econometric result: Marginal effects

**Gender of the household head:** A male-headed household is associated with being 8.5% more likely to be not non-multidimensional poor, 1.2% more likely to be not vulnerable to multidimensional poverty, 0.7% less likely to be moderately multidimensional poor, and 8.9% less likely to be extremely multidimensional poor.

**Marital status of household head:** As the results revealed, a household with a married head was 20% less likely to be severely multidimensional poor, 1% less likely moderately multidimensional poor, 4.2% less likely to be vulnerable to multidimensional poverty, and 17.1% more likely to be not multidimensional poor. This implies that when a household has a married head, it is less likely to fall into multidimensional poverty relative to single (unmarried), divorced, or widowed marital status. The reason for this might be that married households would have responsibilities, thereby reducing unnecessary expenditures. Beyond this, when marriage takes place, there will be an integration of resources, experiences, business ideas, and so on to create a strong economic environment, which will be one way of attacking multidimensional poverty.

**Total income of the household:** A one percent increase in total household income is associated with being 19% more likely to be in the non-multidimensional poor category, 3% more likely to be vulnerable to multidimensional poverty, 3.3% less likely to be moderately multidimensional poor, and 18.7% less likely to be extremely multidimensional poor. When total household income increases, the household’s command over both commodities and business investments will also increase. Thus, as income increases, the probability of falling into multidimensional poverty decreases.

**Family size:** As the number of household members increased by one person, the household became 1.8% more likely to be severely multidimensional poor, 0.3% moderate multidimensional poor, and 0.3% less likely to be vulnerable to multidimensional poverty, and 1.8% less likely to be non-multidimensional poor. The results show that a large number of household members is associated with a higher probability of households falling into a multidimensional poverty circle. When the number of individuals in the house increases, it affects the welfare of the household by increasing living costs. Even if a new child is born, she/he will require care and consume at least adult labor hours each day to look after the child.

**Access to credit**: When the household had access to credit for business operations, the probability of not being multidimensional poor increased by 11.6%, the probability of not being vulnerable to multidimensional poverty increased by 1.6%, the probability of being moderately multidimensional poor decreased by 1.6%, and the probability of being severely multidimensional poor decreased by 12.2%. Access to credit by a household can provide an opportunity to start a profitable business instead of being jobless or remaining low in business activities. In this way, the household’s welfare would be improved, and the probability of falling into multidimensional poverty would decrease.

**Cooperative membership**: When a household was a member of cooperative (s) in its resident, the probability of not being multidimensional increased poor increased by 13%, the probability of not being vulnerable to multidimensional poverty increased by 1.8%, the probability of being moderately multidimensional poor decreased by 1.6%, and the probability of being severely multidimensional poor decreased by 14%. This result is consistent with a study in Nigeria by Adepoju (2018), who found that cooperatives were significant factors in poverty reduction. This variable could reduce MDP by sharing work experience, saving and credit provision, and helping each other members of the cooperative during difficulties. Cooperatives can decrease multidimensional poverty by creating a linkage between poor and rich households that integrates social resources, creating employment and income-increasing opportunities for the poor.

**Female income**: When the household had a female household who was working in income-generating activities, the probability of not being multidimensional increased poor increased by 9.1%, the probability of not being vulnerable to multidimensional poverty increased by 1.2%, the probability of being moderately multidimensional poor decreased by 0.8%, and the probability of being severely multidimensional poor decreased by 9.6%. The results prove that whenever females actively participate in business work, they contribute to the income of the household. Second, they would not get time to give many births, thereby decreasing the number of children in the household. Mohamoud and Bulut (2020) suggest that when female members of a household participate in business activities, the chance of the household being poor can be reduced.

**House ownership**: When the household has its dwelling, the probability of not being multidimensional increased poor increases by 6.4%, the probability of not being vulnerable to multidimensional poverty increases by 0.9%, the probability of being moderately multidimensional poor is decreases by 0.6%, and the probability of being severely multidimensional poor decreases by 6.7%. The result states that households that have their own living house would be less multidimensional poor relative to those that do not their own living house. There are two reasons for this finding. First, owning a house can reduce the cost incurred for rent, and second, the household can construct (build) its own house with standardized qualities.

**Employment types of the household head:** Except for casual wage worker household heads, other types of occupation had a negative marginal effect on multidimensional poverty. However, only permanent income has a significant negative marginal effect on the multidimensional poverty levels of households. When a household has a head who is employed in a permanent income job, it will be 10.1% less likely to be SMP, 0.2% less likely to be moderately multidimensional poor, 1.7% more likely to be non-vulnerable to multidimensional poverty, and 8.6% more likely to be non- multidimensional poor. The reason behind this is because permanently employed household heads have a more stable and reliable income for planning and managing their ways of living than other types of employment. Fields (2019) argued that self-employment in developing countries does not contribute as much to poverty alleviation because self-employment in most developing countries is not backed by the traits of entrepreneurship.

**Education level of the household head:** From education levels of the household head that were considered in this study, only higher education was significant, implying that if the household has a head with a higher diploma, it will be 26.3% less likely to be severely multidimensional poor and 18.9% more likely to be non- multidimensional poor. That is, individuals who have attended higher education levels would have relatively good opportunities to get a good job.

**Saving ratio:** A one percent increase in saving ratio is associated with being 23.5% more likely to be in no multidimensional poverty category, 3.2% more likely to be vulnerable to multidimensional poverty, and 2% less likely to be moderately multidimensional poor, and 24.6% less likely to be extremely multidimensional poor. Saving can reduce the probability of a household being multidimensional poor by providing profit from interest rates and removing waste of resources. More precisely, if a household saves a reasonable percentage of its total income, it will be in a better position for welfare in the future.

## 5. Conclusion and Policy implications

### 5.1 Conclusion

By estimating the multidimensional poverty index of the Debre Tabor city, this study concluded that the majority of households are multidimensional poor. The findings revealed that more than half of the households in this city were deprived of electric energy for cooking purposes, having fixed assets, living in standardized houses, improving sanitation facilities, and receiving health consultations from health careers.

Subgroup decomposition of the multidimensional poverty index indicated that there was a different level of multidimensional poverty in households across their different characteristics. Households with a head with a lower education level were multidimensional poorer than those with highly educated heads. Households with permanently employed heads are also multidimensional richer than households with either self-employed or casual wage workers. Female-headed households were also less multidimensional than their counterparts. This study also concluded that when a household has a single head, divorced and widowed are multidimensional poorer than a household with a married head.

From the regression analysis of socioeconomic factors on multidimensional poverty levels of households, higher education level of the household head, income, saving ratio, access to credit house ownership, female participation in business activities, cooperative membership, permanent employment status of the household head, marital status of the household head (being married), and gender of the household head (being male) were found to be negatively and significantly related to multidimensional poverty levels of households. Family size is positively related to households’ multidimensional poverty levels.

### 5.2 Policy implications

This study had arrived at the following recommendation based on conclusions:

1. **Financial institutions**

The financial sector should create a good environment for receiving savings and providing loans and credit to households in their location. They can achieve this by creating opportunities for credit and creating awareness of how it should be invested. Both private and public financial institutions must provide an interest rate for saving and borrowing with a moderate gap.

2. **Education sector**

The education office should not leave anyone behind in school. It should create awareness in households to send school-aged children to schools. In addition, in collaboration with non-governmental organizations, the sector must support children who cannot maintain school materials. Furthermore, they must provide basic writing and reading education for adults.

3. **Health offices and family planning sector**

These government administration sectors must predominantly work on birth control. They can do this by clarifying the scientific importance of contraceptives. They can also provide a model for how children grow in greater quality of mind and physical settings when there is an appropriate distance between their birthday.

4. **Social and Labor affair sector**

Officials in this sector must pay great attention to the contract between daily wage workers and their employers. This can be done in a way that prepares strict rules on wages, updates the rules in line with the change in living costs, and follows up on whether the rules are being respected. Otherwise, a measurement must be taken.

5. **Municipality of Debre Tabor city**

This should make the environment conducive for households to have neat and livable housing. That is, they can make land available for households to construct their own houses or construct condominiums and redistribute them to households.

6. **Households**

Households should allow and appreciate their female members to participate in business activities. The can do this by giving the females equal chances to education, and they should allow them to participate in household decisions. Households should also actively participate in existing cooperatives, and they should form new cooperatives for their residents. Household heads should not be divorced.

## Author contributions

All three authors contributed significantly to the writing of this manuscript. They worked on the conception, design, data collection tool development, data collection, entry, analysis, and manuscript writing in consultation as a team.

## Ethical consideration

The study authors collected data from household heads of Debre Tabor University which were selected on random basis. Before data collection respondents gave verbal consent to give responses for the questions related to multidimensional poverty variables. The investigators assured them that their identity will be kept confidential. As a result, the identity of any of the respondents was not mentioned in any part of the study and the dataset. The participants of the study are adults (household heads) and as there is no experiment on this study, participants are just respondents; not subjects of the study. In addition, we have got approval from college research ethics review committee “Research and community service Review Committee”. We confirm that the date of ethical approval is 13-September-2023 Ref No: DTU/CBE-4/07/2023. Therefore, the study is based on scientific ethics.

We have taken “verbal consent” from all participants of the study.

The name of the college level review committee is: “Research and community service Review Committee”. The University level Institutional Review board has decentralized some review tasks to college level research and community service review committees to facilitate Ethical review. This is particularly true in case when study units are adults and just respondents if the study doesn’t include experimental design. In such a case, verbal consent is assumed to be sufficient to take information using questionnaire. Thus, this duty is assigned to college level research and community service review committee. The committee approved verbal consent in line with the power vested to it.

Verbal consent form was applied to this study as respondents are adults and the study doesn’t related to experimental design in human being.

The researchers approached each respondent first by explaining the purpose of the study and asked the same verbally whether it is ok to proceed or he/she is not willing to participate. Then, questionnaire is given to those who accepted willfully to participate.

## Data Availability

The dataset used for this study is available as follows: Harvard Dataverse: Analyzing Multidimensional Poverty and Its Determinants in Ethiopia: A Micro-level Analysis at Debre Tabor City Administration, Dataset https://dataverse.harvard.edu/dataset.xhtml?persistentId=doi:10.7910/DVN/OVWVOU. This project contains:
•
MPI_dataset.xlsx Data are available under the terms of the Creative Commons Zero “No rights reserved” data waiver (CC0 1.0 Public domain dedication). The dataset used for this study is available as follows: Harvard Dataverse: Analyzing Multidimensional Poverty and Its Determinants in Ethiopia: A Micro-level Analysis at Debre Tabor City Administration, Dataset https://doi.org/10.7910/DVN/OVWVOU. This project contains:
• MPI_Questionnaire and Long tables.docx Data are available under the terms of the Creative Commons Zero “No rights reserved” data waiver (CC0 1.0 Public domain dedication).
