# Supplementary material for: Analyzing Multidimensional Poverty and Its Determinants in Ethiopia: A Micro-level Analysis at Debre Tabor City Administration
Source: F1000Res. 2025 Feb 13;14:203. [Version 1] doi: 10.12688/f1000research.159139.1 (PMC12317311; doi:10.12688/f1000research.159139.1)
Supplement: Peer review history

# Reviewer report: Reviewer response for version 1

**DOI:** 10.5256/f1000research.174825.r396601

**Published:** 2025-08-01

**Authors:** Badreddine Amina

**Affiliations:** 1 M'hamed Bougara Boumerdes University, Bourmerdes, Algeria

This article presents a rigorous analysis of multidimensional poverty in Debre Tabor, Ethiopia, using the Alkire-Foster method and ordered logistic regression. It identifies energy use, income, and living standards as key poverty drivers, with female-headed, single, and informally employed households being most vulnerable. The methodology is robust, and the findings are well-supported and policy-relevant. The paper offers clear recommendations on education, employment, and credit access. Minor improvements in language clarity and literature contextualization are needed. Overall, it is a valuable and timely contribution deserving indexing after minor revisions.

Is the work clearly and accurately presented and does it cite the current literature?

Yes

If applicable, is the statistical analysis and its interpretation appropriate?

Yes

Are all the source data underlying the results available to ensure full reproducibility?

Yes

Is the study design appropriate and is the work technically sound?

Yes

Are the conclusions drawn adequately supported by the results?

Yes

Are sufficient details of methods and analysis provided to allow replication by others?

Yes

Reviewer Expertise:

econometrics, poverty, startups, sustainability, tourism

I confirm that I have read this submission and believe that I have an appropriate level of expertise to confirm that it is of an acceptable scientific standard.

## References

[1] : Determinants of Household Poverty: Identification Using Logistic Regression. *SocioEconomic Challenges* .2024;8(1) : 10.61093/sec.8(1).78-89.2024 78-89 10.61093/sec.8(1).78-89.2024

# Reviewer report: Reviewer response for version 1

**DOI:** 10.5256/f1000research.174825.r366535

**Published:** 2025-02-28

**Authors:** Emmanuel A Onsay

**Affiliations:** 1 University of the Philippines Los Baños, Laguna, Philippines

Overall, the paper presents a well-structured and insightful discussion. With some enhancements and modifications, it has the potential to be highly relevant for policy and decision-making.

 The title could be more precise and impactful if revised to:

 "Multidimensional Poverty and Its Determinants in Ethiopia: A Micro-Level Study of Debre Tabor City Administration."

 The abstract is concise but would benefit from additional sentences to strengthen the arguments. Expanding on the study's key findings, methodology, and significance will enhance its clarity and impact. Additionally, citing the sampling design and the basis for selecting the sample would provide greater methodological transparency.

 For the keywords, consider making them more direct and closely related to your study to improve their relevance and searchability.

 In the introduction, the paper is well-supported with citations, effectively integrating multiple sources to establish a strong research foundation. The definition of poverty is comprehensive, moving beyond economic deprivation to highlight its social, political, and environmental dimensions. The inclusion of recent statistical data from sources such as EDHS, UNDP, and OPHI strengthens the argument and provides a clear contextual background for Ethiopia.

 However, there are areas for improvement. Some sentences are lengthy and complex, making them harder to follow. Breaking them down into more concise statements would improve clarity and readability. The logical progression of ideas is well-structured, transitioning from a broad global perspective to Ethiopia’s specific case. However, smoother transitions between sections would further enhance the flow. A brief connecting sentence before introducing Ethiopia’s multidimensional poverty data would create a more seamless narrative.

 Maintaining terminology consistency is also important. For example, the discussion of poverty measurement methods in the final paragraph should clearly connect to the concepts introduced earlier. Ensuring uniformity in terminology will help prevent confusion and maintain coherence.

 Grammar and syntax adjustments will further refine the clarity of the text. For instance, the sentence:

*"They are embarrassed and furious and are unable to maintain their regular social circles and friendships."*

 Could be rephrased as:

*"They often experience embarrassment and frustration, which hinder their ability to maintain social connections."*

 Additionally, while statistical data is well-incorporated, a concluding sentence summarizing the significance of these percentages would reinforce their importance.

 Regarding the literature review (RRL), paragraphs should contain more than 10 sentences each to ensure depth and coherence in discussions.

 For methodology, you may find the following references useful in enhancing your approach:

 Ref: 1, 2

 To improve the scaling and implications of your results, consider expanding the analysis to include measures of poverty incidence, gap, and severity, provided the data allows for such computations.

 Finally, the policy implications section should be presented in paragraph form to ensure a structured and comprehensive discussion. Clearly articulating how the findings inform policy and decision-making will strengthen the study’s real-world relevance.

 By addressing these refinements, your paper will achieve greater clarity, coherence, and impact.

Is the work clearly and accurately presented and does it cite the current literature?

Yes

If applicable, is the statistical analysis and its interpretation appropriate?

Yes

Are all the source data underlying the results available to ensure full reproducibility?

Yes

Is the study design appropriate and is the work technically sound?

Yes

Are the conclusions drawn adequately supported by the results?

Partly

Are sufficient details of methods and analysis provided to allow replication by others?

Yes

Reviewer Expertise:

NA

I confirm that I have read this submission and believe that I have an appropriate level of expertise to confirm that it is of an acceptable scientific standard.

## References

[1] : Measuring the unmeasurable multidimensional poverty for economic development: Datasets, algorithms, and models from the poorest region of Luzon, Philippines. *Data Brief* .2024;53: 10.1016/j.dib.2024.110150 110150 10.1016/j.dib.2024.110150 38379883 PMC10877178

[2] : Measuring the Unmeasurable: Decomposing Multidimensional Rural Poverty and Promoting Economic Development in the Poorest Region of Luzon, Philippines. *Societies* .2024;14(11) : 10.3390/soc14110235 10.3390/soc14110235
